# Six-years survival and predictors of mortality after CABG using cold vs. warm blood cardioplegia in elective and emergent settings

**DOI:** 10.1186/s13019-015-0384-9

**Published:** 2015-12-04

**Authors:** Mohamed Zeriouh, Ammar Heider, Parwis B. Rahmanian, Yeong-Hoon Choi, Anton Sabashnikov, Maximillian Scherner, Aron-Frederik Popov, Alexander Weymann, Ali Ghodsizad, Antje-Christin Deppe, Axel Kröner, Ferdinand Kuhn-Régnier, Jens Wippermann, Thorsten Wahlers

**Affiliations:** 1 Department of Cardiothoracic Surgery, Heart Center, University of Cologne, Kerpener Str. 62, 50937 Cologne, Germany; 2 Department of Cardiothoracic Transplantation and Mechanical Circulatory Support, Royal Brompton & Harefield NHS Foundation Trust, Harefield Hospital, Harefield, Middlesex UK; 3 Department of Cardiac Surgery, University of Heidelberg, Heidelberg, Germany; 4 Heart and Vascular Institute, Pennstate Hershey, Philadelphia, PA USA

## Abstract

**Background:**

The aim of this study was to determine whether intermittent warm blood cardioplegia (IWC) is associated with comparable myocardial protection compared to cold blood cardioplegia (ICC) in patients undergoing elective vs. emergent CABG procedures.

**Methods:**

Out of 2292 consecutive patients who underwent isolated on-pump CABG surgery using cardioplegic arrest either with ICC or IWC between January 2008 and December 2010, 247 consecutive emergent patients were identified and consecutively matched 1:2 with elective patients based on gender, age (<50 years, 50–70 years, >70 years) and ejection fraction (<40 %, 40–50 %, >50 %). Perioperative outcomes and long-term mortality were compared between ICC and IWC strategies and predictors for 30-day mortality and perioperative myocardial injury were identified in both elective and emergent subgroups of patients.

**Results:**

Preoperative demographics and baseline characteristics, logistic Euroscore, CPB-time, number of distal anastomoses and LIMA-use were comparable. Aortic cross clamp time was significantly longer in the IWC-group regardless of the urgency of the procedure (*p* = 0.05 and *p* = 0.015 for emergent and elective settings). There were no significant differences regarding ICU-stay, ventilation time, total blood loss and need for dialysis. The overall 30-day, 1-, 3- and 6-year survival of the entire patient cohort was 93.7, 91.8, 90.4 and 89.1 %, respectively, with significantly better outcomes when operated electively (*p* < 0.001) but no differences between ICC and IWC both in elective (*p* = 0.857) and emergent (*p* = 0.741) subgroups. Multivariate analysis did not identify the type of cardioplegia as a predictor for 30-day mortality and for perioperative myocardial injury. However, independent factors predictive of 30-day mortality were: EF < 40 % (OR 3.66; 95 % CI: 1.79–7.52; *p* < 0.001), atrial fibrillation (OR 3.33; 95 % CI: 1.49-7.47; *p* < 0.003), peripheral artery disease (OR 2.51; 95 % CI: 1.13–5.55; *p* < 0.023) and COPD (OR 0.26; 95 % CI: 1.05–6.21; *p* < 0.038); predictors for perioperative myocardial infarction were EF < 40 % (OR 2.04; 95 % CI: 1.32–3.15; *p* < 0.001), preoperative IABP support (OR 3.68; 95 % CI: 1.34-10.13; *p* < 0.012), and hemofiltration (OR 3.61; 95 % CI: 2.22–5.87; *p* < 0.001).

**Conclusion:**

Although the aortic cross clamp time was prolonged in the IWC group our results confirm effective myocardial protection under IWC, regardless of the urgency of the procedure. We suggest that intermittent warm cardioplegia in emergent CABG setting is a low-cost alternative and safe. It is associated with similar long-term outcomes both in elective and emergent settings compared to intermittent cold cardioplegia.

## Background

The concept of myocardial protection during cardiac surgery have been already described in detail in the 1950s by Bigelow, who proposed to use hypothermia as a strategy to protect the myocardium in heart surgery [1]. In this respect, a reduction in myocardial oxygen consumption up to 80 % can be achieved by lowering the blood temperature up to 20 °C. However, the disadvantage of the isolated hypothermia without an additional usage of cardioplegia results in an incomplete electromechanical cardiac arrest with ventricular fibrillation. Therefore, subsequent studies by Gay and Ebert support the use of potassium-rich solution for the induction of diastolic cardiac arrest by depolarization [2]. In addition, further development of blood cardioplegia by Follette and Buckberg as a composition of blood and crystalloid solution improved myocardial protection enormously [2, 3]. Finally, the next breakthrough was the development of normothermic blood cardioplegia in which Calafiore has set new standards in myocardial protection during cardiac surgery [4, 5].

Currently, a large variety of techniques for myocardial protection is available in cardiac surgery. Most surgical centers in the USA and west european countries preferably apply blood cardioplegia as several studies indicated superiority of blood cardioplegia over crystalloid solutions [6]. Nevertheless, the debate over the optimal cardioplegia strategy, the way of application and temperature is still under discussion [7]. Moreover, other additional potentially cardioprotective effects on ischemic myocardium are discussed in the literature [8]. In our institution intermittent blood cardioplegia is used in coronary artery bypass grafting (CABG) patients and is administered antegradely either as intermittent normothermic cardioplegia (IWC) according to Calafiore’s warm blood cardioplegia or intermittent hypothermic cardioplegia (ICC) according to Buckberg’s cold blood cardioplegia. Nowadays, the IWC as a simple and cost-effective method is in widespread use throughout the world. Whereas several studies have been focused on differences and similarities in blood cardioplegia in elective CABG patients, there has been a lack of evidence in terms of applicability of different cardioplegia in emergent settings. The aim of the present study was to critically examine both myocardial protection strategies in patients undergoing emergent CABG and patients receiving elective CABG.

## Methods

### Patients

This was a retrospective study at the Heart Center of the University of Cologne with a cohort consisting of a total of 2292 patients who underwent isolated CABG from January 2008 to December 2010. Of them 247 consecutive patients were treated as an emergency and were consecutively matched 1:2 with elective coronary bypass patients based on age (<50, 50–70 or >70 years), gender and ejection fraction (<40 %, 40–50 % or >50 %). Due to several missing values in terms of preoperative patient characteristics and differences regarding baseline data between elective and emergent patients, the number of elective patients with exact matching criteria reached 448. In total, we explored two groups with a total number of 695 patients, subsequently dividing them depending on the blood cardioplegia strategy, with 176 patients (25.3 %) receiving ICC versus 506 patients (72.8 %) receiving IWC. A total of 13 patients were excluded after matching due to missing variables of interest. A subgroup analysis for each urgency status was performed. Furthermore, factors associated with early mortality as well as independent factors predictive of 30-day mortality in elective and emergent settings were evaluated.

### Operation technique

CABG was standardized in terms of surgical technique and perioperative management. In brief, all operations were performed with cardiopulmonary bypass (CPB) via median sternotomy. CPB was implemented with arterial cannulation of the ascending aorta and venous drainage which was connected to the right atrium with a two-stage venous cannula. A root suction was always placed in the aortic root. Left and/or right internal mammary arteries (LIMA and RIMA) and/or venous grafts were used as graft material. Distal anastomoses were connected during the total aortic cross clamp time. Proximal anastomoses were performed within reperfusion time using a partial clamping technique of the ascending aorta. Finally, after decannulation, insertion of chest drains and sternal closure intubated patients were transferred to the intensive care unit (ICU).

### Application of cardioplegia

In terms of cardioplegia protocol in our institution, the so called „cold induction“ and „cold reinfusion“ Buckberg cardioplegia is normally used. The components are supplied separately in two bottles (400 mL and 100 mL) and are first mixed together when needed. The induction cardioplegia solution contains: 400 mL of high potassium concentration with KCl 34 mmol, THAM 17.45 mmol and NaCl 16.12 mmol; following substances are added with the second 100 mL bottle: citric acid 0.45 mmol x H_2_O, sodium citrate 2.60 mmol x 2 H_2_O, sodium hydrogen phosphate 0.47 mmol x 2 H_2_O and glucose 92.42 mmol x H_2_O. For the reinfusion cardioplegia there are also two bottles that are first mixed when needed: 400 mL low potassium concentration of KCl 16.90 mmol, THAM 18.30 mmol and NaCl 16.90 mmol. As with induction cardioplegia, additional substances are added from the 100 mL bottle: citric acid 0.48 mmol, sodium citrate 2.73 mmol, sodium hydrogen phosphate 0.49 mmol and glucose 96.93 mmol. This concentrated solution is diluted in a ratio 1:4 with blood to provide oxygen, the desired pH and osmolarity. The cardioplegic induction solution with high potassium concentration is delivered over 4 min to initiate cardiac arrest whereas the following reinfusion solution with lower potassium concentration is delivered over 2 min to maintain cardiac arrest every 15–20 min.

Cardioplegia was delivered into the aortic root immediately after cross clamping ascending aorta and re-infused into vein grafts after completion of the distal anastomosis in patients with severely obstructed or occluded coronary arteries. In the ICC group, cardiac arrest was initiated by cold induction using a mixture of the patient’s own blood with the crystalloid Buckberg solution (Dr. Franz Köhler Chemie GmbH, Germany) at a ratio of 1 to 4 and blood flow rate of 200–300 mL/min. Patients were receiving cold (4–6 °C) blood cardioplegia for 4 min aiming for a potassium target dose of 20 mmol/L. In the ICC group the body temperature was routinely cooled down to 34 °C. According to our institutional policy there was no additional topical cooling. While the aorta was cross clamped, intermittent re-infusions for 2–4 min each with ICC were applied at intervals of 15–20 min. Despite recommendations by the Buckberg protocol warm terminal reperfusion (‘hot shot’) was not infused prior to aortic unclamping in our center. Over the study period a total of 8 surgeons were performing elective and emergent CABG. Whereas 3 surgeons were advocates of Buckberg cardioplegia (ICC), other 5 surgeons were proponents of Calafiore cardioplegia (IWC). There were no surgeons who had used both types of cardioplegia dependent on clinical factors. Also, as it is a retrospective analysis, the choice of cardioplegia could not be influenced.

According to our institutional policy the body temperature using IWC was maintained between 36 °C and 37 °C. In the IWC group, infusion of normothermic (37 °C) oxygenated blood was administered to the aortic root using a standardized setup. A syringe pump was connected to the extracorporeal circulation containing a modified mixture of 30 mL of KCl (2 mmol/L) and 10 ml of MgSO4 (2 mmol/L). Cardiac arrest was induced by a syringe pump with the flow rate of 150 mL/h at a blood flow rate of 200–300 mL/min for approximately 2 min with the aim to achieve potassium concentration of 20 mmol/L and magnesium target dose of 5 mmol/L. In cases when cardiac arrest was not achieved immediately, an additional bolus injection of 3–4 ml cardioplegia was infused. Thereafter, the cardioplegic solution was repeatedly administered every 15 min or after completion of each distal anastomosis, whereby the flow rate of the syringe pumps was reduced (120-90-60 mL/h) according the protocol of Calafiore et al. [5].

### Data collection

All data were collected from our institutional database. The Electronic Patient Record of our department contains all patient data, such as demographics, preoperative risk factors, intra- and postoperative data. Moreover, the preoperative medication, especially the use of aspirin and clopidogrel and their discontinuation were recorded. In addition, laboratory values were obtained from the institutional database.

### Definitions

Thirty day mortality was defined as death of any cause within 30 days after CABG. Perioperative myocardial infarction (PMI) was defined as the combination of the two following criteria proposed by Liakopoulos et al. [8] higher than fivefold increase in creatine kinase MB (CK-MB >120 U/L) including CK-MB fraction range of 6–25 % and an increase in Troponin T over 1.5 ng/mL within 72 h after surgery. Further studies have also shown that those laboratory tests are qualified for the diagnostics of PMI in patients undergoing cardiac surgery, especially Troponin T [10–12].

### Statistical analysis

Statistical analysis was performed using the IBM SPSS statistics version 19 (SPSS Inc., Chicago, IL, USA). Continuous variables were expressed as mean ± standard deviation and categorical variables given as absolute values and percentages. A comparison of the two groups was done using ANOVA for continuous variables and Chi square test or Fisher exact test for categorical variables. *P* values of < 0.05 were considered statistically significant. Univariate analysis was executed to demonstrate associations between outcome and perioperative variables. Binary logistic regression analysis was performed to detect independent predictors for 30-day mortality. Kaplan-Meier Survival analysis was performed with the view to presenting long-term outcomes of the entire patient cohort as wall as comparing outcomes in subgroups.

## Results

Patient demographics and preoperative baseline characteristics are listed in Table 1. There were no statistically significant differences between ICC and IWC groups except for proportion of patients on regular statin medication (66.7 % in the ICC group vs. 80.7 % in the IWC group, *p* = 0.035) in the emergent subgroup and with the presence of left main disease with more than 50 % stenosis (34.5 % in the ICC group vs. 23.5 % in the IWC group, *p* = 0.026) in the elective subgroup. Patients were comparable in terms of past medical history, cardiac risk factors, preoperative medication and logistic Euroscore.

**Table 1:** Preoperative data, emergent vs. elective CABG devided in ICC and IWC

	Emergent CABG					Elective CABG				
	ICC		IWC			ICC		IWC		
	n	%	n	%	p-Wert	n	%	n	%	p-Wert
Age > 70	23	36.5	63	36.2		45	39.8	117	35.2	
Female gender	14	22.2	36	20.7	0.857	24	21.2	71	21.4	1.000
HT	49	27.8	142	28.2	0.450	105	59.7	295	58.3	0.279
Pulm. HT	6	9.5	11	6.5	0.410	4	3.5	12	3.6	1.000
Hyperlipidemia	46	73.0	12	65.1	0.276	92	81.4	261	78.6	0.592
Diab. mellitus	16	25.4	58	33.5	0.269	38	33.6	134	40.4	0.220
BMI > 30	14	22.2	43	24.7	0.734	42	37.2	98	29.5	0.159
AF	14	23.7	33	22.2	0.857	5	4.4	20	6.0	0.641
3 V-CAD	50	79.4	136	78.2	1.000	97	85.8	274	82.5	0.467
LMS	31	49.2	81	47.1	0.883	39	34.5	78	23.5	0.026
COPD	4	6.3	20	11.6	0.332	15	13.3	35	10.5	0.490
PAD	10	15.9	31	17.9	0.847	20	17.7	52	15.7	0.658
Smoker	30	47.6	91	52.9	0.556	46	40.7	137	41.3	1.000
Crea >2	5	7.9	11	6.3	0.770	2	1.8	11	3.3	0.317
CVA	8	12.7	31	18.0	0.429	12	13.3	57	17.2	0.377
Beta-blocker	48	76.2	139	81.3	0.462	86	76.1	274	82.5	0.165
Statin	42	66.7	138	80.7	0.035	92	81.4	256	77.1	0.359
Antiplatel.	55	91.7	156	94.5	0.532	26	23.0	54	16.3	0.120
EF < 40 %	23	36.5	48	27.6		33	29.2	97	29.2	
Euroscore Logistic	29.82 ± 27.14		25.09 ± 23.95		0.214	7.26 ± 10.91		7.20 ± 9.48		0.953

Preoperative Data: emergent- vs. elective CABG devided in intermittent cold cardioplegia (ICC) and intermittent warm cardioplegia (IWC) accordingto Buckberg and Calafiore, respectively. Arterial fibrillation (AF), 3 vessel-coronary artery disease (3 V-CAD), cerebrovascular accident(CVA), Hypertension (HT), left main stenosis (LMS), peripherial artery disease (PAD)

Table 2 represents intra-operative patient data for both groups. In the emergent subgroup, patients receiving IWC had longer cross clamp time (41.1 ± 13.9 vs. 35.4 ± 12.2 min, *p* = 0.005), whereas patients receiving ICC were associated with significantly longer administration time (5.9 ± 2,2 vs. 5.0 ± 2.7 min, *p* = 0.017) and amount (329 ± 155 vs. 18.5 ± 5.6 ml, *p* < 0.001) of cardioplegia compared to IWC group. Similar associations were also found within the elective subgroup. In terms of intraoperative data within both subgroups there were no statistically significant differences in CPB, operation and, reperfusion time, and the usage of IMA. Groups were also comparable regarding phosphodiesterase-III-inhibitor usage, and the need for hemofiltration.

**Table 2:** Intraoperative data, emergent vs. elective CABG devided in ICC and IWC

	Emergent CABG					Elective CABG				
	ICC		IWC			ICC		IWC		
	n	%	n	%	p-Wert	n	%	n	%	p-Wert
AOX (min)	35.38 ± 12.16		41.05 ± 13.91		0.005	39.92 ± 12.65		43.96 ± 15.85		0.015
CBP (min)	81.81 ± 37.75		90.50 ± 38.68		0.121	77.96 ± 25.20		82.93 ± 32.25		0.138
OP-time (min)	182.03 ± 46.10		198.52 ± 64.67		0.064	184.39 ± 46.24		201.31 ± 95.14		0.092
Number of distal grafts	2.94 ± 0.76		3.09 ± 0.91		0.245	3.01 ± 0.84		2.93 ± 0.78		0.386
LIMA use	58	92.1	158	90	1.000	107	94.7	315	94.9	1.000
RIMA use	7	11.1	27	15.5	0.530	28	24.8	83	25.0	1.000
Reperfusion time (min)	34.87 ± 22.05		38.35 ± 27.35		0.366	28.54 ± 12.69		30.72 ± 17.55		0.225
Cardioplegia time (min)	5.91 ± 2.22		5.01 ± 2.68		0.017	7.03 ± 3.05		5.08 ± 2.73		0.000
Cardioplegia in (mL)	329.02 ± 155.43		18.54 ± 5.6		0.000	370 ± 125.60		19.15 ± 21.51		0.000
Hemofiltration	22	34.9	48	27.6	0.333	13	11.5	37	11.1	1.000
PDI (Corotrop)	21	34.4	63	36.4	0.877	13	15.9	75	22.6	0.143

Intraoperative patients data: emergent- vs. elective CABG devided in intermittent cold cardioplegia (ICC) and intermittent warm cardioplegia (IWC). Aortic clamp time (AOX), cardiopumonary bybass (CBP), LIMA (left internal mammary artery), RIMA (right internal mammary artery), phospodiesterase inhibitor (PDI)

**Fig. 1:**
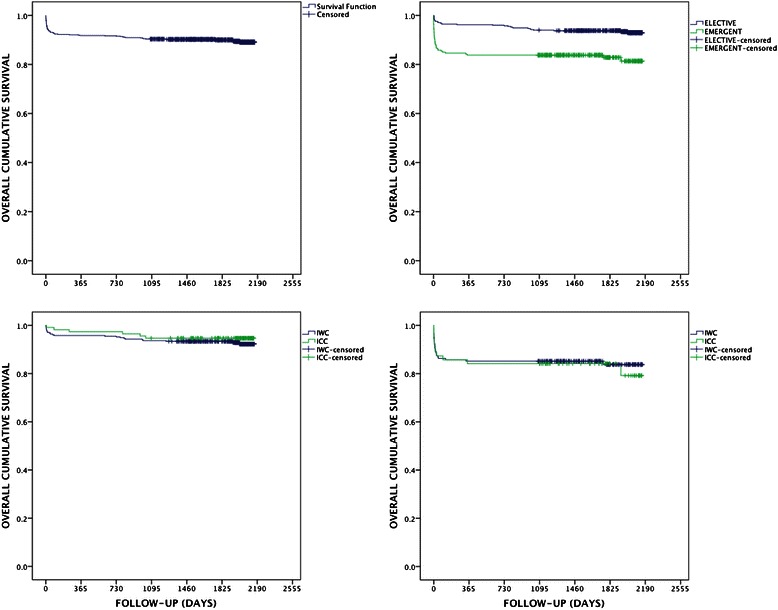
Long-term overall cumulative survival of the entire patient cohort with 30-day, 1-, 3- and 6-year survival of 93.7, 91.8, 90.4 and 89.1 %, respectively (left upper graph); significantly better outcomes when operated electively (*p* < 0.001, right upper graph); no differences between ICC and IWC groups both in elective (*p* = 0.857, left lower graph) and emergent (*p* = 0.741, right lower graph) subgroups

Postoperative patients’ characteristics are presented in Table 3. The mean number of platelet units transfused in the elective subgroup was 0.62 ± 1.43 in the ICC group and 0.31 ± 0.72 in the IWC group (*p* = 0.003). The 30-day all cause mortality in the elective subgroup tended to be increased in the IWC group with 2 % in contrast to 0 % in ICC group, however it did not reach statistical significance (*p* = 0.072). Similarly, in the emergent subgroup, the 30-day mortality was 5,1 % in the ICC group vs. 4,2 % in the IWC group not reaching statistical significance. During cardiac surgery in the emergent subgroup 10.8 % patients of the ICC group received an intra-aortic balloon pump (IABP) compared to 6.5 % of the IWC group also not reaching statistical significance (*p* = 0.077). Further outcome variables, such as PMI, length of hospital and ICU stay, ventilation duration, extracorporeal membrane oxygenation (ECMO), dialysis, inotropic support, blood transfusion and total blood loss were also not statistically different. Long-term outcomes in terms of overall cumulative survival are presented in Figure 1. The overall 30-day, 1-, 3- and 6-year survival of the entire patient cohort was 93.7, 91.8, 90.4 and 89.1 %, respectively, with significantly better outcomes when operated electively (*p* < 0.001) but no differences between ICC and IWC both in elective (*p* = 0.857) and emergent (*p* = 0.741) subgroups.

**Table 3:** Postoperative data, emergent vs. elective CABG devided in ICC and IWC

	Emergent CABG					Elective CABG				
	ICC		IWC			ICC		IWC		
	*n*	%	*n*	%	*p*-value	*n*	%	*n*	%	*p*-value
30 day mortality	9	5.1	31	4.7	0.661	0	0.0	10	2.0	0.072
PMI	12	6.8	28	U	0.563	13	7.4	31	6.1	0.584
ICU(d)	6.44 ± 11.80		5.73 ± 5.70		0.536	3.83 ± 4.15		4.01 ± 4.50		0.708
Hospital (d)	13.19 ± 12.77		13.53 ± 9.39		0.825	12.98 ± 7.21		13.05 ± 6.20		0.979
Duration of ventilation (h)	109.85 ± 171.87		76.85 ± 150.32		0.194	44.45 ± 168.25		30.97 ± 73.03		0.24/
IABP										
- preop	10	5.7	19	3.8	0.370	1	0.6	2	0.4	1.000
- intraop	19	10.8	33	6.5	0.077	2	1.1	8	1.6	1.000
- postop	3	1.7	9	1.8	1.000	1	0.6	5	1.0	1.000
ECMO	5	2.8	11	2.2	0.770	0	0.0	0	0.0	
Blood transfusion										
- RBC	7.76 ± 12.92		5.96 ± 7.30		0.183	3.93 ± 5.79		3.45 ± 5.07		0.401
- FFP	4.43 ± 6.8		2.79 ± 4.21		0.210	1.50 ± 3.95		1.19 ± 3.29		0.411
- Platelets	1.46 ± 1.45		1.05 ± 1.10		0.220	0.62 ± 1.43		0.31 ± 0.72		0.003
Total blood loss (ml)	1537.81 ± 1049.81		1400.17 ± 1019.14		0.425	1085.00 ± 715.66		962.03 ± 622.91		0.084
Dialysis	3	1.7	21	4.2	0.142	3	1.7	11	2.2	1.000
Inotropie support > 24 h	14	8.9	48	10.1	0.858	23	14.6	56	11.7	0.390
Inotropie support > 48 h	17	10.8	58	12.2	0.734	12	7.6	52	10.9	0.274

Postoperative patients data: emergent- vs. elective CABO devided in intermittent cold cardioplegia (ICC) and Intermittent warm cardioplegia (IWC). Extra Corporeal Mrrubianr Onygrri.ition (ECMO), inti.i aortic balloon pump countrrpuKation (IABP), prfimycK.irdi.il infarction (PMI)

Univariate regression analysis was executed (Tables 4 and 5), in order to show possible associations of early clinical outcome with determined variables. There were no associations found between each clinical endpoint and the application of IWC or ICC.

**Table 4:** Univariate analysis, primary endpoint: 30-days mortality

Univariate analysis, primary endpoint: 30-days mortality (*n* = 45)							
Variable	Yes		No		O.R.	95 %-C.I.	*p*-value
	*n*	%	*n*	%			
Age > 70 y	17	37.8	28	6.4	1.04	(0.56 – 1.95)	0.875
Female	13	28.9	32	5.9	1.55	(0.79 – 3.03)	0.192
HT	35	79.5	9	9.9	0.55	(0.26 – 1.22)	0.163
pulmonal. HT	7	16.3	36	5.5	4.44	(1.81 – 10.89)	0.003
HLP	21	47.7	23	13.4	0.27	(0.15 – 0.51)	<0,001
Diab. mellitus	18	40.0	27	6.1	1.18	(0.64 – 2.19)	0.632
BMI > 30	13	28.9	32	6.5	1.01	(0.52 – 1.96)	1.000
AF	15	34.1	29	4.8	4.60	(2.34 – 9.03)	<0,001
3 V–CAD	32	71.1	13	10.1	0.54	(0.27 – 1.05)	0.074
LMS	20	44.4	25	5.4	1.63	(0.89 – 3.00)	0.141
COPD	9	20.0	36	5.8	2.13	(0.99 – 4.62)	0.080
PAD	15	33.3	30	5.2	2.62	(1.36 – 5.03)	0.007
Smoker	22	50.0	22	5.7	1.27	(0.59 – 2.33)	0.531
Crea >2	6	14.0	37	5.7	4.32	(1.66 – 11.24)	0.007
Renal insuff. preop. Crea <2	23	51.1	22	4.7	2.25	(1.23 – 4.14)	0.013
CVA	6	13.6	38	6.6	0.79	(0.33 – 1.91)	0.333
Beta–blocker	27	62.8	16	11.8	0.38	(0.20 – 0.73)	0.005
Statin	22	51.2	21	13.6	0.27	(0.14 – 0.51)	<0,001
Antiplatelet	29	67.4	14	3.6	2.90	(1.50 – 5.59)	<0,001
EF < 40 %	30	66.7	15	3.1	5.36	(2.82 – 10.21)	<0,001
EF 40–50 %	8	17.8	37	7.0	0.70	(0.32 – 1.53)	0.466
EF >50 %	7	15.6	38	10.4	0.19	(0.08 – 0.43)	<0,001
Euroscore > =6	40	95.2	2	0.6	16.98	(4.07 – 70.85)	<0,001
IABP preop.	6	13.3	39	5.9	3.68	(1.43 – 9.45)	0.013
NYHA III + IV	34	75.6	11	3.3	2.96	(1.48 – 5.94)	0.002
Emergent CABG	34	75.6	11	2.5	6.43	(3.15 – 12.76)	<0,001
preop. myocardial infarction	22	50.0	22	4.4	3.26	(1.76 – 6.06)	<0,001
Time between angiography and operation:							
<3d	27	65.9	14	3.2	4.41	(2.26 – 8.60)	<0,001
<24 h	26	63.4	15	3.1	5.79	(2.99 – 11.24)	<0,001
OP–time after 08.00 pm	6	13.3	39	6.2	1.57	(0.64 – 3.86)	0.292
ICC cardioplegia	10	23.8	32	6.3	0.89	(0.43 – 1.86)	0.857
IWC cardioplegia	31	73.8	11	6.2	0.99	(0.48 – 2.00)	1.000
AoX < 80 min	37	88.1	5	35.7	0.11	(0.03 0.33)	0.001
AoX > 60 min	6	14.3	36	5.9	1.39	(0.57 – 3.43)	0.444
AoX >45 min	13	31.0	29	6.3	0.91	(0.46 – 1.78)	0.866
CPB >180 min	10	22.7	34	5.0	37.82	(12.25 116.80)	<0,001
CPB > 120 min	20	45.5	24	3.9	9.36	(4.85 – 18.04)	<0,001
OP–time > 180 min	33	73.3	12	3.9	2.47	(1.25 – 4.88)	0.008
Reperfusion time > 60 min	17	39.5	26	4.1	13.40	(6.57 – 27.34)	<0,001
Hemofiltration	27	60.0	IS	3.2	8.54	(4.53 – 16.10)	<0,001
PDI (Corotrop)	24	54.5	20	2.9	3.71	(2.00 – 6.90)	<0,001
PMI	36	80.0	9	1.7	14.71	(6.92 – 31.26)	<0,001
ICU > 3d	29	69.0	13	3.3	3.28	(1.67 – 6.43)	<0,001
ICU > 5d	19	45.2	23	4.6	2.52	(8.83 – 34.82)	0.006
ICU > 7d	16	38.1	26	4.8	2.59	(1.35 – 4.97)	0.005
ICU > 10d	12	28.6	30	5.1	3.13	(1.53 – 6.38)	0.003
Hospital stay > 15 d	10	22.7	34	6.3	1.07	(0.51 – 2.21)	0.851
Ventilation time >48 h	23	62.2	14	2.6	11.23	(5.54 – 22.73)	<0,001
IABP preop.	27	60.0	18	2.9	15.91	(8.25 – 30.67)	<0,001
ECMO	15	33.3	30	4.4	107.83	(29.61 – 392.72)	<0,001
Blood transfusion	43	100.0	0	0.0	0.92	(0.90 – 0.94)	0.068
RBC Unit >5	31	68.9	14	2.7	8.48	(4.39 – 16.39)	<0,001
Total blood loss >1l	19	65.6	10	2.7	2.62	(1.20 – 5.73)	0.020
Total blood loss >2l	12	41.4	17	3.0	6.17	(2.82 – 13.52)	<0,001
Dialysis	12	26.7	33	5.0	8.08	(3.77 – 12.30)	<0,001
Inotropie support >48 h	26	89.7	3	0.8	12.19	(3.65 40.70)	<0,001
ACT > 150 sec	10	41.7	14	3.1	4.33	(1.85 – 10.10)	<0,001
Pneumonia	10	22.7	34	5.1	12.39	(5.19 – 29.62)	<0,001
Wound infection	3	6.8	41	6.5	0.77	(0.23 – 2.57)	1.000
Operative re–exploration	13	29.5	31	4.8	6.91	(3.34 – 14.31)	<0,001

Pre-_r_intra- and postoperative data in univariate analysis. The nominal variables are shown in absolute and percentage figures. Cerebrovascular accident (CVA), 3 vessel coronary artery disease (3-V CAD), left main stenosis (LMS), Red blood cells (RBC)

**Table 5:** Univariate analysis, primary endpoint: Perimyocardial Infarction (PMI)

Univariate analysis, primary endpoint: PMI (*n* = 175)							
Variable	Yes		No		O.R.	95 %-C.I.	*p*-value
	*n*	%	*n*	%			
Age > 70 years	66	(37.7)	109	(24.8)	1.05	(0.74 – 1.50)	0.786
Female	36	(20.6)	139	(25.4)	0.94	(0.62 – 1.44)	0.832
HT	141	(S1.0)	33	(35.3)	0.54	(0.34 – 0.85)	0.013
pulmonal HT	14	(8.1)	158	(24–2)	2.19	(1.08 – 4.44)	0.040
HLP	122	(70.1)	52	(30.2)	0.71	(0.48 – 1.04)	0.085
Diab. mellitus	63	(36.0)	112	(25.4)	0.98	(0.69 – 1.40)	0.928
BMI >30	50	(2S.6)	125	(25.3)	0.99	(0.68 – 1.44)	1.000
AF	39	(22.9)	131	(21.6)	3.46	(2.14 – 5.58)	<0,001
3 V–CAD	140	(SO.O)	35	(27.1)	0.88	(0.57 – 1.36)	0.575
LMS	73	(42–5)	100	(21.8)	1.67	(1.17 – 2.38)	0.005
COPD	20	(H–4)	155	(25.2)	1.04	(0.61 – 1.79)	0.890
PAD	39	(22.3)	136	(23.7)	1.57	(1.02 – 2.41)	0.048
Smoker	84	(48.3)	90	(23.4)	1.23	(0.87 – 1.73)	0.253
Crea >2	14	(8–5)	151	(23.3)	3.08	(1.45 – 6.52)	0.006
Renal insuff. preop Crea <2	78	(44.6)	97	(20.8)	1.97	(1.38 – 2.80)	<0,001
CVA	36	(20.7)	138	(23.9)	1.47	(0.95 – 2.28)	0.098
Beta–blocker	127	(73.4)	46	(33.3)	0.58	(0.39 – 0.87)	0.011
Statin	117	(57.6)	55	(96.4)	0.49	(0.33 – 0.72)	<0,001
Antiplatelet	127	(75.1)	42	(10.9)	6.19	(4.17 – 9.19)	<0,001
EF < 40 %	SO	(46.0)	94	(19.3)	2.66	(1.85 – 3.80)	<0,001
EF 40–50 %	51	(29.3)	123	(23.1)	1.54	(1.05 – 2.27)	0.038
EF >50 %	43	(24.7)	131	(35.7)	0.27	(0.19 – 0.40)	<0,001
Euroscore > =6	138	(S2.6)	29	(9.9)	5.13	(3.31 – 7.93)	<0,001
IABP preop	25	(144)	149	(22.6)	12.25	(5.20 – 28.88)	<0,001
NYHA 111 + IV	116	(55.3)	59	(17.9)	2.12	(1.49 – 3.04)	<0,001
Emergent CABG	125	(71.4)	50	(11.2)	8.16	(5.55 – 11.99)	<0,001
Time between angiography and operation							
<3d	100	(65.8)	52	(11.9)	6.61	(4.45 – 9.82)	<0,001
<24 h	92	(50.5)	52	(12.4)	8.77	(5.83 – 13.19)	<0,001
OP–time after 08.00 pm	35	(20.0)	60	(22.2)	4.22	(2.49 – 7.15)	<0,001
ICC	47	(28.0)	121	(23.9)	1.16	(0.79 – 1.72)	0.477
IWC	120	(71.4)	48	(27.1)	0.84	(0.57 – 1.23)	0.363
AoX < 80 min	164	(95.5)	5	(42.9)	0.43	(0.15 1.25)	0.122
AoX > 60 min	17	(10.0)	153	(25.0)	0.88	(0.50 – 1.56)	0.777
AoX > 45 min	53	(31.2)	117	(25.4)	0.90	(0.62 – 1.30)	0.574
CPB > 180 min	13	(7.5)	161	(23.8)	20.83	(4.65 – 93.29)	<0,001
CPB > 120 min	42	(24.1)	132	(21.3)	5.00	(3.03 – 8.26)	<0,001
OP–time > 180 min	111	(54 2)	62	(20.3)	1.75	(1.23 – 2.51)	0.002
Reperfusion time > 60 min	35	(20.5)	136	(21.2)	10.83	(5.47 – 21.43)	<0,001
Hemofiltration	59	(39.4)	106	(18.6)	5.49	(3.64 – 8.29)	<0,001
PDI (Corotrop)	81	(53.2)	92	(18.1)	3.64	(2.51 – 5.26)	<0,001
30–days mortiality	36	(20.6)	139	(21.4)	14.71	(6.92 – 31.25)	<0,001
ICU > 3d	118	(72.0)	46	(11.8)	5.27	(3.58 – 7.76)	<0,001
ICU > 5d	36	(52.4)	78	(15.6)	5.18	(3.54 – 7.59)	<0,001
ICU > 7d	72	(43.9)	92	(17.1)	5.30	(3.55 – 7.93)	<0,001
ICU > 10d	46	(32.0)	118	(19.9)	4.87	(3.03 – 7.83)	<0,001
Hospital stay > 15 d	55	(32.0)	117	(21.7)	2.11	(1.42 – 3.11)	<0,001
Ventilation time >48 h	72	(47 4)	80	(14.6)	14.49	(8.86 – 23.71)	<0,001
IABP perioperativ	83	(47.4)	92	(15.0)			<0,001
ECMO	18	(10.3)	157	(23.2)			<0,001
Blood transfusion	156	(97.6)	4	(7.5)	4.37	(1.55 – 12.30)	0.001
EK >5	84	(48.3)	90	(17.1)	5.04	(3.44 – 7.37)	<0,001
Total blood loss >11	91	(62.8)	52	(14.8)	2.83	(1.93 – 4.14)	<0,001
Total blood loss > 21	38	(26.2)	107	(18.8)	4.43	(2.69 – 7.29)	<0,001
Dialysis	27	(15.4)	148	(22.6)	7.12	(3.58 – 14.14)	<0,001
Inotropie support >48 h	113	(77.4)	33	(9.1)	6.69	(4.35 – 10.28)	<0,001
CardioAssist	105	(60.0)	70	(12.0)	109.93	(49.16 – 245.83)	<0,001
ACT > 150 sec.	26	(21.1)	97	(21.3)	1.69	(1.01 – 2.83)	0.048
Pneumonia	17	(9.8)	156	(23.4)	6.95	(2.94 – 16.41)	<0,001
Wound infection	15	(8.7)	158	(25.0)	1.02	(0.55 – 1.89)	1.000
Operative re–exploration	25	(14.5)	148	(23.1)	3.33	(1.86 – 5.97)	<0,001

Pre-,intra- and postoperative data in univariaten analysis. The nominal variables are shown in absolute and percentage figures, cerebro vascular accident (CVA), hypertension (HT), 3 vessel-coronary artery disease (3 V-CAD), left main stenosis (LMS), perimyocardial infarction (PMI), red blood cells (RBC)

In order to detect independent preoperative predictors for 30-day mortality and PMI binary logistic regression analysis was performed (Table 6 and 7) using only preoperative variables. Multivariate analysis revealed that EF < 40 % was the strongest independent predictor for 30-day mortality (OR 3.66; 95 %-CI 1.79-7.52; *p* < 0.001), followed by atrial fibrillation, PAD and anti-platelet drugs not suspended at least 5 days before surgery (Table 6). In contrast, three independent predictor variables showed a protective influence on 30-day mortality, namely 3-vessel-disease (OR 0.36; 95 %-CI 0.16-083; *p* = 0.016), preoperative statin therapy and dyslipidemia. The strongest independent predictor for PMI appeared to be preoperative anti-platelet drug therapy (OR 4.79; 95 %-CI 3.10-7.40; *p* < 0.001), followed by IABP, hemofiltration, EF < 40 %, atrial fibrillation and cerebrovascular disease (Table 7). Preoperative statin therapy tended to be a borderline predictor with a protective influence on PMI (OR 0.66; 95 %-CI 0.41-1.08; *p* = 0.101).

**Table 6:** Multivariate analysis, primary endpoint: 30-days mortality

Multivariate analysis, primary endpoint: 30-days mortality (*n* = 45)			
Variable	O.R.	95 %-C.I.	*p*-value
HLP	0.39	(0.19 – 0.79)	0.009
AF	3.33	(1.49 – 7.47)	0.003
3 V–CAD	0.36	(0.16 – 0.83)	0.016
COPD	0.26	(1.05 – 6.21)	0.038
PAD	2.51	(1.13 – 5.55)	0.023
Statin	0.38	(0.18 – 0.80)	0.010
Antiplatelet.	1.99	(0.95 – 4.17)	0.070
EF <40 %	3.66	(1.79 – 7.52)	<0,001
Variable used in multivariable analysis:			
Female			
HT			
pulm. HT			
HLP			
Diab. mellitus			
AF			
3 V–CAD			
LMS			
COPD			
PAD			
Smoker			
Crea >2			
Renal insuff. preop Crea <2			
CVA			
Beta-blocker			
Statin			
Antiplatelet.			
EF <40 %			
IABP preop			

*AF* Atrial fibrillation, *CVA* cerebrovascular accident, *HLP* Hyperlipidemia, *HT* Hypertension, *PAD* peripherial artery disease, *LMS* left main stenosis

**Table 7:** Multivariate analysis, endpoint: PMI

Multivariate analysis, endpoint: PMI (*n* = 175)			
Variable	O.R.	95 %-C.I.	*p*-value
AF	1.79	(0.98 – 3.27)	0.060
CVA	1.68	(0.98 – 2.89)	0.059
Statin	0.66	(0.41 – 1.08)	0.101
Antiplatelet	4.79	(3.10 – 7.40)	<0,001
EF < 40 %	2.04	(1.32 – 3.15)	0.001
IABP preap	3.68	(1.34 – 10.13)	0.012
Hemofiltration	3.61	(2.22 – 5.87)	<0,001
Variable used in multivariable analysis:			
art. HT			
pulm. HT			
AF			
LMS			
HLP			
PAD			
Crea >2			
Renal insuff. preop Crea <2			
CVA			
Beta–blocker			
Statin			
Antiplatelet			
EF <40 %			
IABP preop.			
OP-time after 08:00 pm			
Hemofiltration			

*AF* Atrial fibrillation, *CVA* cerebro vascular accident, *HLP* Hyperlipidemia, *HT* Hypertension, *PAD* peripherial artery disease, *LMS* left main stenosis

### Analysis of cause of death

In the IWC and ICC emergent CABG group 8 patients vs. 5 patients died after acute myocardial infarction with postcardiotomy ECMO, 2 of them had cerebrovascular events, 4 patients vs. 1 patient died due to severe sepsis with multiorgan failiure, 2 patients vs 0 patients with mesenterial ischemia and laparotomy, 1 patients vs. 0 patients with LV and LA thrombus in association with heparin induced thrombocytopenia and 6 patients vs. 3 patients died postoperatively after CPR due to electromechanical dissociation and low-output syndrome, respectively.

On the other hand, in the IWC and ICC elective CABG group 3 patients vs. 0 patients died after pericardial tamponade or pulmonary emboli with postcardiotomy ECMO, 3 patients vs. 0 patients died due to severe sepsis and multiorgan failiure, 1 patient vs 0 patients after mesenterial ischemia and laparotomy in association with sepsis and 3 patients vs. 0 patients postoperatively after CPR due to electromechanical dissociation and low-output syndrome, respectively.

## Discussion

As several clinical studies have shown superior outcomes when using blood cardioplegia, this type of myocardial protection has been increasingly implemented in most centers including our department [6, 13]. So far, most prior research has been particularly focused on cold and warm blood cardioplegia in elective CABG patients [14, 15]. Also the differences between crystalloid and cold blood cardioplegia have been largely debated in previous research [16, 17]. In order to fill the gap in terms of analysis of differences in cold and warm blood cardioplegia in emergent setting, we conducted our study with particular attention to emergent CABG patients analysing the impact of different intermittent blood cardioplegia application. Previous research has indicated that there were no significant differences between antegrade and retrograde application of blood cardioplegia in terms of mortality and myocardial infarction [14, 18]. Due to easier technical aspects it has become the standard of care to administer antegrade blood cardioplegia for CABG procedures in our institution. In this study we analyzed different cardioplegia strategies in emergent compared to elective CABG patients. The intraoperative patient data showed similar results referring to CPB, operation, and reperfusion time, usage of IMA and the need for hemodialysis. However, it is interesting to note that even though the aortic cross clamp time was significantly longer in the IWC group there were no differences in PMI. In a recent study it was even suggested that cold blood cardioplegia seems to be associated with more harmful effects on the myocardium in CABG patients when compared to warm blood cardioplegia [19]. In contrast, another study by Liakopoulos et al. demonstrated that cold blood cardioplegia according to Buckberg provided superior myocardial protection in terms of 30-day mortality, cardiac death and PMI in patients requiring prolonged aortic cross clamp time during cardiac surgery [9]. However, it is very doubtful as to whether a meaningful comparison can be made between CABG and non-CABG surgery procedures precisely because severe coronary stenosis may impair cardioplegic delivery leading to a different delivery of each solution beyond the stenosis.

Another study with prospective randomized design which was published two decades ago showed strong evidence that warm blood cardioplegia was superior to cold blood cardioplegia in CABG patients in terms of PMI [20]. As it was a randomized study, there were several exclusion criteria, such as patients with renal failure, or emergent patients who were not able to sign informed consent. In order to present a real world experience, we did not intend to exclude any cases, analyzing consecutive patients including those requiring preoperative mechanical ventilation or patients with renal failure. Moreover, an emergent subgroup was created and matched with consecutive patients from the elective subgroup in order to provide the full analysis in patients with different risk profiles. Additionally, compared to the study by Warm Heart Investigators where cardioplegia was given antegradely and/or retrogradely depending on surgeon’s preference this potential bias is not present in our study as cardioplegia was always administered antegradely [20]. Similar to our report, another study by Calafiore et al. investigated 500 consecutive CABG patients who received IWC compared to ICC [5]. However, the blood cardioplegia in that study was given antegradely in all cases, the main difference to our study is that Calafiore et al. only included elective CABG patients. Interestingly, Calafiore et al. found that the outcome in the IWC group was superior to the ICC group in terms of requirement for circulatory assistance, IABP, and incidence of myocardial infarction and cerebrovascular accidents. In another prospective randomized trial Franke et al. also showed a significantly improved myocardial protection in the IWC group compared to ICC group in elective setting [15]. Surprisingly, there is only one experimental animal study which showed that IWC is superior to ICC in ischemic myocardium [21]. Despite the fact that the concept of myocardial protection in the beginning of cardiac surgery was recommended with introduction of hypothermic crystalloid cardioplegia it has been continuously adjusted and improved by pharmacological additives, blood cardioplegia and different application techniques [22]. Despite the fact that warm and cold cardioplegia resulted in similar 30-days and long-term mortality in our study, there has been a substantial amount of research showing that warm cardioplegia reduces several adverse post-operative events [7, 23]. However, totally different results were found in a meta-analysis with a total of 41 randomized controlled trials including 5879 patients comparing warm vs. cold cardioplegia. This study demonstrated no statistically significant differences in the incidences of clinical events [24]. Hence, our results seem to support this theory that there are no statistically significant differences in using cold vs. warm blood cardioplegia in emergent CABG patients particularly those with aortic cross clamp time less than 60 min according to the mean cross clamp time results of our study. Additionally, we performed a multivariate analysis which further showed that the type cardioplegia is not an independent predictor for PMI or 30-day mortality independent of the procedure urgency.

In terms of our additional findings suggesting preoperative risk factors for PMI the strongest independent predictor appeared to be preoperative use of anti-platelet drugs also supported by Micelli et al. demonstrated previously [25]. Our further findings on predictors of early mortality, such as decreased EF of less than 40 %, AF and PAD are also consistent with previous research [26, 27]. Whereas van Straten et al. found evidence that PAD is associated with an increased risk for long-term mortality, our findings suggested that PAD is also an independent predictor for short-term mortality [28]. Finally, our data support that preoperative statin treatment may have protective effects on mortality similarly to recent studies by Kulik et al. and Girerd et al. [29, 30] and prevents cardiovascular complications by pleiotropic effects [31]. Nevertheless, optimal mortality prediction should still be performed using established scoring systems [32, 33]. Our study is also in consistence with the fact that statins may reduce cytokine release and neutrophil adhesion via a nitric oxide-mediated mechanism and improve postoperative myocardial perfusion of bypassed areas [34, 35].

## Conclusions

Summarizing, there are no clinically significant differences in using cold and warm blood cardioplegia in emergent CABG patients including long-term outcomes regardless of the urgency status. Decreased EF, AF and PAD remain main predictors for 30-day mortalitiy whereas preoperative use of statins is associated with protective effects. Warm blood cardioplegia and normothermic systemic perfusion offer safe myocardial protection strategies in emergent CABG patients and IWC is a cost-effective alternative. Therefore, we suggest to use warm blood cardioplegia antegradely in elective and emergent CABG procedures regardless of the emergent status.

### Limitations

This is a retrospective study with consecutive elective and emergent patients. Even though we consecutively matched the emergent cohort 1:2 to a control elective cohort to avoid selection bias there is still a limitation in interpreting the results due to the use of cardioplegia depending on surgeons’ preferences. There were surgeons who routinely used cold blood cardioplegia and surgeons who routinely used warm blood cardioplegia which may have influenced cross clamp times in IWC and ICC groups.
